# Comparative Evaluation of the Artefacts Index of Dental Materials on Two-Dimensional Cone-beam Computed Tomography

**DOI:** 10.1038/srep26107

**Published:** 2016-05-17

**Authors:** Fusong Yuan, Litong Chen, Xiaofei Wang, Yong Wang, Peijun Lyu, Yuchun Sun

**Affiliations:** 1 Centre of Digital Dentistry, Peking University School and Hospital of Stomatology & National Engineering Laboratory for Digital and Material Technology of Stomatology & Research Centre of Engineering and Technology for Digital Dentistry of Ministry of Health, 22 Zhongguancun Nandajie, Haidian District Beijing, 100081, China

## Abstract

The aim of the study was to propose the artefact index on cone-beam computed tomography (CBCT) images of clinical prosthodontics materials, and to compare the effect of the artefacts on CBCT image clarity of normal oral tissues. Seven spheres of different materials were secured on the centre of a resin baseboard, respectively, and four human molars *in vitro* were placed at 10 mm front, back, left and right of the sphere. The board was scanned using CBCT with the same setting. 10 tomographic images from each of the seven data sets with clear artefacts was selected. The grayscale measuring tool of Photoshop software was used to measure the grayscale (G0) within the boundary of tomographic image and the grayscales of the streaky artefacts that were 1 mm and 20 mm outside the circular boundary (G1 and G2). The arc length, L1, of the circular boundary with artefacts was measured; the circumference, L2, was calculated. The artefact index, A, was determined as (G1/G0) × 0.5 + (G2/G1) × 0.4 + (L2/L1) × 0.1. The artefact index A can comprehensively represent the effect of artefacts on CBCT image clarity for oral tissue.

Cone beam computed tomography (CBCT) has entered oral and maxillofacial region as an alternative for conventional CT and a valuable addition to two-dimensional radiographic modalities for diagnosis, treatment planning and follow-up. Enabling high-resolution three-dimensional (3D) imaging of hard tissues at a low radiation dose, CBCT has proven to be useful in many dental areas such as the assessment of dento-alveolar pathology, maxillofacial surgery, orthodontics, implantology, and endodontics^1^,^2^.

A common problem in CBCT based imaging of the oral cavity is artefacts^3^,^4^,^5^,^6^, which reduce the assessment quality of the oral cavity. These are commonly caused by dental restorations made of metal alloys. Metal, as a high-density object, induces a much higher attenuation coefficient of passing radiation compared to materials with a low density. Thus, X-ray radiation is drastically attenuated because of the effect of these objects, resulting in data distortion of the corresponding projection and artefacts in reconstructed images. These artefacts induced by metal implants are all referred to as metal artefacts^7^. Metal artefacts are one of several types of Artefacts found in all types of CT imaging^8^,^9^,^10^. Metallic restorations, crowns, brackets, and implants affect the image quality of a reconstructed CT image due to effects such as beam hardening, scatter, quantum noise, and photon starvation^5^,^11^. Beam hardening results in the increase of the mean beam energy after passing through the metal object due to the predominant absorption of low-energy X-rays. Scatter refers to X-rays deflecting from their original path but still reaching the detector, leading to faulty projection data. Quantum noise increases the graininess of the image due to a contamination of the detector signal. Furthermore, complete absorption (i.e., starvation) of all photons along certain beam paths may occur. These effects result in different types of image deterioration, ranging from bright streaks radiating from the metallic object to darkening of areas in its vicinity and even the complete loss of gray values between adjacent metallic objects^12^. As a result, regions of interest for diagnosis, planning or follow-up are not properly visualized. This affects the accuracy of dentists’ judgment on dental illness or treatment outcome. The effects of metallic objects in CBCT imaging are similar to those observed in conventional CT. It has been reported by many authors that the diagnostic image quality of head and neck CT or CBCT is hampered by the presence of metallic objects in the dental area^13^,^14^,^15^,^16^,^17^,^18^. Dental materials are classified into metallic materials and non-metallic-materials. However, there is currently no non-metallic-materials Artefacts that be reported and standardized evaluation method available to quantify the effect of metallic materials and non-metallic-materials artefacts.

The aim of the study was to propose the artefacts index on CBCT images of dental restoration materials, and to compare the effect on CBCT image clarity of normal oral tissues.

## Results

**Figure 1:**
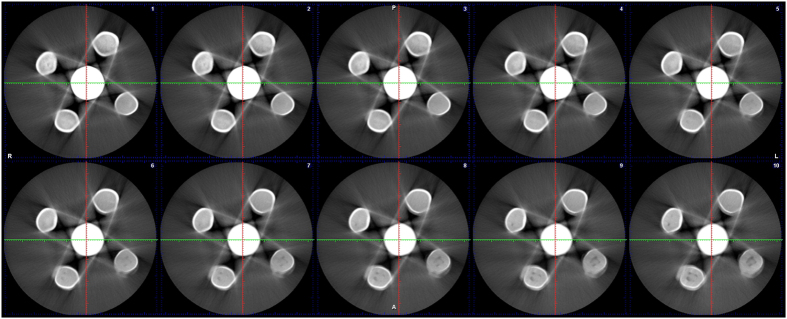
Ten two-dimensional CBCT images of one dental material with clear artefacts.

**Figure 2:**
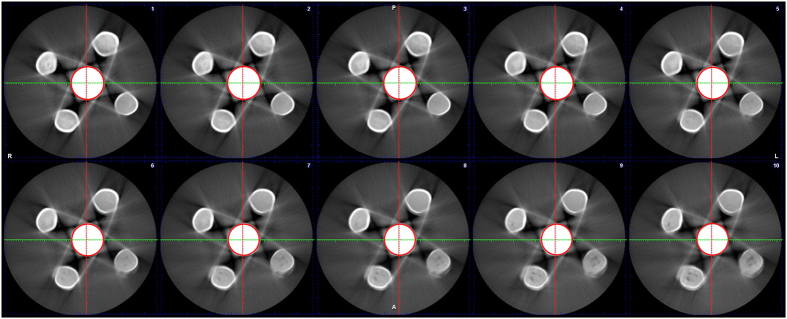
The sphere boundary on ten two-dimensional CBCT images of one dental material with clear artefacts.

10 two-dimensional CBCT images with relatively clear sphere artefacts were displayed (Fig. 1), and the sphere boundaries were shown (Fig. 2). The measured G0 values within the boundary are shown in Table 1. The mean value of G0 was 255.

**Figure 3:**
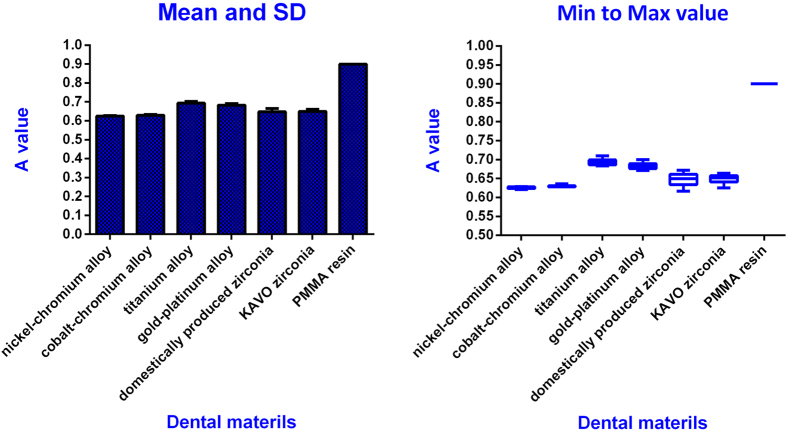
Statistical Description of the A values of seven dental materials.

The A values of 7 dental materials were within 1; the maximum was 0.900, the minimum was 0.625, the A values from small to large were ordered as follows: nickel-chromium alloy (0.625), cobalt-chromium alloy (0.631), domestically produced zirconia (0.648), KAVO zirconia (0.649), gold-platinum alloy (0.683), titanium alloy (0.694), and poly (methyl methacrylate) (PMMA) resin (0.900) (Table 2) (Fig. 3). Using one-way ANOVA analysis, the A values differed significantly among the different dental materials (P < 0.05) (Table 3).

The A value was inversely proportional to the influence on two-dimensional tomography image clarity. In the study, the least influence was PMMA resin, and the biggest impact was nickel-chromium alloy.

## Discussion

Artefacts are an important factor affecting oral and maxillofacial CBCT image quality. The method to evaluate image artefacts was proposed in the study based on image pixel grayscale values. The obtained two-dimensional CBCT images were grayscale images. Each pixel had a fixed grayscale value, which refers to the depth of colour in black-and-white images, and normally ranges from 0 to 255, with white being 255 and black being 0. In the selected two-dimensional CBCT images of dental materials, the grayscale value of the normal sphere position, G0, was 255, while the values of the surrounding tissues were lower. The grayscale difference between the boundary and surrounding tissues was higher in images with clearer boundaries, which could be more easily identified by the naked eye, and vice versa. For the boundary regions that could not be easily identified by the naked eye, we provide the evaluation method for image artefacts based on pixel grayscale differences. The artefacts and clarity evaluation method proposed herein for two-dimensional CBCT images of prosthodontics can be completed by using the commonly used two-dimensional image processing software, Photoshop CS6, with easy processes and calculations. It is simple and practical, can obtain the correct boundary locations, and quantitatively evaluate the artefacts of dental materials. This method could assist dentists in the quantitative identification and comparison of artefacts in prostheses, and can provide some guidance for the clinical choice of appropriate dental materials. But, there are still some limitations about the method.

The container used in this experiment mainly aimed to mimic the oral space. According to a previous study^19^, dry objects can induce artefacts when CT scanning is performed in air. Therefore, we filled the container with water in this experiment and conducted CBCT of the dental materials in water. This method not only mimicked the moist environment inside the mouth, but also avoided the artefacts due to dry air exposure, in order to produce images that can better reflect the effects in real clinical settings. However, the container is not good to simulate the bone. In the future study, we will select a material that is close to the density of the bone tissue, and print out the standard head with 3D system, which can simulate the human oral environment more realistically.

The type of CBCT and the different exposure protocols can change the artefacts. In the study, we used one CBCT device with a single protocol that selected normally in clinical practice in order to reduce the influence of different kinds of CBCT devices and different scanning parameters on experimental results. But at present, there are many types of CBCT devices and multiple exposure protocols that can be selected. Large differences are seen for the CBCT devices with regard to the tube voltage peak, tube current-time product field of view size, and reconstructed voxel size. In order to make the research more practical in clinic, different kinds of CBCT and different scanning parameters were chosen for the further study, in order to improve comprehensiveness and practicability of the study.

The shapes and sizes of the inserts would change the artefacts in the CBCT images. In order to avoid the impact of irregular morphology and size of restoration on the results, uniform diameter balls were used in the study. But, dental restorations are generally the irregular bodies of different sizes. With the purpose of making the study more reflecting the clinical situation, the balls will be replaced by different restorations, such as the crown, bridge, implants and removable partial denture.

In conclusion, this method based on grayscale differences of image pixels on two-dimensional CBCT image artefacts of dental prosthesis can provide good quantitative evaluation of artefacts and clarity about dental materials. Therefore, we believe that this method is more reliable compared with identification with the naked eye. For its limitations, we plan to continue performing research on this subject to improve and supplement this method in order to produce a more accurate and reliable artefacts evaluation method.

## Methods

### Equipment and materials

7 solid sphere with 15 mm diameter were prepared form the following dental materials: nickel-chromium alloy (Ni_28_-Cr_24_, Ni_28_ 65%, Cr_24_ 22.5%), cobalt-chromium alloy (Co_27_-Cr_24_, Co_27_ 61%, Cr_24_ 26%), titanium alloy (Ti_22_-Al_13_-V_23_, Ti_22_ 90%, Al_13_ 6%, V_23_ 4%), or gold-platinum alloy (Au_79_-Pt_78_, Au_79_ 85%, Pt_78_ 15%); domestically produced zirconia (ZrO_2_); KAVO zirconia (ZrO_2_); or poly(methyl methacrylate) (PMMA) resin; A CBCT (New Tom VG, Verona, Italy) was used to measure the Hounsfield Unit value; A 25 cm diameter × 20 cm tall water-filled plastic cylinder was used as the head phantom; A light-cured resin stationary plate was use to fix the solid sphere and human molar *in vitro*; the medical imaging software (Mimics 10.01, Leuven, Belgium) and image processing software (Adobe Photoshop CS3, California, USA) were used to analyse the datasets.

### Collection and pre-processing of the tooth samples

4 human intact first molars that were freshly extracted at the Hospital of Stomatology in Peking University were collected. The study was approved by the Bioethics Committee of the Stomatological Hospital of Peking University (Beijing, China; No. PKUSSIRB-201522044; Date: 07/08/2015). All of the experimental protocols and procedures were approved by the licensing committee and performed in accordance with the approved guidelines and regulations. The patients were informed that the extracted teeth would be used in the *in vitro* study, and informed consent was obtained from all of the subjects. An ultrasonic scalar was used to remove dental calculi and soft tissues from the surface of the removed teeth, which were then rinsed with physiological saline.

### Production of two-dimensional CBCT images of prosthodontics materials

The resin sphere was secured on the stationary baseboard, while 4 human molars were separately placed at 10 mm on front, back, and to the left and right of the sphere. The stationary board was fixed at the bottom of the water-filled round plastic bucket. The position of the bucket was the same as that of a human head during normal CBCT scanning. The exposure factors of CBCT were set for 110 KV, 20 mA, 18-second rotation time with no added filtration. And the CBCT scan was performed with a rotation of 360° for data acquisition. The limits of the imaging area consisted of a cylinder 20 cm in height and 25 cm in diameter. With the position of the board fixed, the resin sphere was individually replaced with spheres of the other six materials, and the scanning was repeated in the same setting. A total of seven sets of digital imaging and communications (DICOM) in medicine data were obtained and imported to the MIMICS software, to produce a 520-layer hierarchical display. 10 layers with obvious artefacts were selected for quantitative evaluation and analysis of artefacts. The same layers was selected in the seven sets of data.

### Determination of sphere boundaries of prosthodontics materials on two-dimensional CBCT images

The obtained two-dimensional tomographic images were imported into Photoshop CS6 software. The part of the arc in the sphere image boundary that had no artefacts was used to determine the centre and the radius, and to construct the correct circular boundary. The grayscale, G, of 10 randomly selected points within the circle was measured. The mean value represented the normal grayscale (G0) of the crown.

### Quantitative evaluation of artefacts around prosthodontics materials on two-dimensional CBCT images

The grayscale measuring tool of Photoshop was used to measure the grayscale of the streaky artefacts outside the boundary that were 1 mm and 20 mm from the circular boundary, i.e., G1 and G2, respectively. The arc length, L1, of the circular boundary that contained artefacts was measured; the circumference, L2, was calculated. The hardening artefacts index, A, was determined as A = (G1/G0) × 0.5 + (G2/G1) × 0.4 + (L2/L1) × 0.1. The A values of the seven materials were calculated. Consequently, 70 values (7 × 10) were measured. The mean values for each group were calculated. The results were analysed with SPSS 20.0 (IBM Corporation, Armonk, NY, USA). One-way analysis of variance (ANOVA) was used to evaluate the variance of artefacts index among different dental materials.

## Additional Information

**How to cite this article**: Yuan, F. *et al.* Comparative Evaluation of the Artefacts Index of Dental Materials on Two-Dimensional Cone-beam Computed Tomography. *Sci. Rep.* **6**, 26107; doi: 10.1038/srep26107 (2016).

## Figures and Tables

**Table 1:** Normal grayscale measurements of prosthodontics materials on two-dimensional CBCT images ( 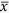 = 255, s = 0).

Marker point	1	2	3	4	5	6	7	8	9	10
Grayscale	255	255	255	255	255	255	255	255	255	255

**Table 2:** Statistical description of A value on the two-dimensional CBCT images of the seven prosthodontics materials.

	N	Mean	Std. Deviation	Std. Error	95% Confidence Interval for Mean		Minimum	Maximum
					Lower Bound	Upper Bound		
1	10	0.62557	0.002818	0.000891	0.62355	0.62758	0.621	0.629
2	10	0.63103	0.003478	0.001100	0.62854	0.63352	0.628	0.636
3	10	0.69383	0.008502	0.002689	0.68775	0.69992	0.683	0.710
4	10	0.68307	0.008354	0.002642	0.67710	0.68905	0.671	0.700
5	10	0.64816	0.016847	0.005327	0.63611	0.66021	0.617	0.672
6	10	0.64912	0.011634	0.003679	0.64080	0.65744	0.625	0.664
7	10	0.90000	0.000000	0.000000	0.90000	0.90000	0.900	0.900
Total	70	0.69011	0.089876	0.010742	0.66868	0.71154	0.617	0.900

Note: Material 1. Nickel-Chromium alloy; 2. Cobalt-Chromium alloy; 3. Titanium alloy; 4. Gold-Platinum alloy; 5. Domestically Produced Zirconia; 6. KAVO Zirconia; 7. PMMA resin.

**Table 3:** ANOVA of A value.

	Sum of Squares	df	Mean Square	F	Sig.
**Between Groups**	0.552	6	0.092	1108.160	0.000
**Within Groups**	0.005	63	0.000		
**Total**	0.557	69			
